# Differences in Mortality of Pre-Weaned and Post-Weaned Juvenile European Hedgehogs (*Erinaceus europaeus*) at Wildlife Rehabilitation Centres in the Czech Republic

**DOI:** 10.3390/ani13030337

**Published:** 2023-01-17

**Authors:** Gabriela Kadlecova, Sophie Lund Rasmussen, Eva Voslarova, Vladimir Vecerek

**Affiliations:** 1 Department of Animal Protection and Welfare and Veterinary Public Health, Faculty of Veterinary Hygiene and Ecology, University of Veterinary Sciences Brno, 612 42 Brno, Czech Republic; 2 Wildlife Conservation Research Unit, The Recanati-Kaplan Centre, Department of Biology, University of Oxford, Tubney House, Abingdon Road, Tubney, Abingdon OX13 5QL, UK; 3 Department of Chemistry and Bioscience, Aalborg University, Fredrik Bajers Vej 7H, DK-9220 Aalborg, Denmark

**Keywords:** hoglet, weaning, rescue centre, release

## Abstract

**Simple Summary:**

Wildlife rehabilitation centres contribute to the conservation of wildlife by caring for sick, injured and orphaned animals that would not survive in the wild without human help, and releasing healthy animals back into the wild in appropriate habitats. A total of 4388 European hedgehogs, identified as pre-weaned from normally timed litters (NL PRE), post-weaned from normally timed litters (NL POST), pre-weaned from late litters (LL PRE) and post-weaned from late litters (LL POST) were admitted to 27 wildlife rehabilitation centres in the Czech Republic in the period from 2011 to 2020. Where the outcome of rehabilitation care was known, young admitted before natural weaning were associated with a high mortality rate, especially in those from late litters. Among the four groups, the juveniles of the NL POST category experienced the lowest mortality (14%) with the highest release rate (86%). In contrast, LL PRE experienced the highest mortality (46%) with the lowest release rate (54%).

**Abstract:**

Previous research from several European countries has indicated that the European hedgehog (*Erinaceus europaeus*) is in decline. Wildlife rehabilitation centres contribute toward the protection of debilitated hedgehogs, including the young. Based on data from 27 wildlife rehabilitation centres, the mortality rate and the release rate of juvenile hedgehogs were evaluated depending on whether they were from normally timed litters (admitted from April to September) or from late litters (admitted from October to March). A total of 4388 juvenile European hedgehogs were admitted to wildlife rehabilitation centres in the Czech Republic from 2011 to 2020. The number of post-weaned young from late litters admitted (28%) did not differ from the number of pre-weaned young from late litters (29%). Where the outcome was known, young from late litters had the highest mortality rate (46%) in the year of admission. The release rate was the highest in post-weaned young from normally timed litters (86%). Further research should focus on the definition of optimal care and treatment of the underlying causes for admission of juvenile hedgehogs. The reproductive strategy (the timing of litters) of European hedgehogs under the climatic conditions of the Czech Republic affects the chance of survival of young at wildlife rehabilitation centres and likely also in the wild.

## 1. Introduction

The western European hedgehog (*Erinaceus europaeus*) is an insectivore that inhabits a large part of Europe [1,2]. It is a species that displays true hibernation. It is, therefore, essential for hedgehogs to reach an optimal body condition to survive hibernation [3,4,5]. The length of hibernation and the timing of its ending may differ depending on how long the winter period lasts in a given area in which hedgehogs are found. Hibernation is known to be shorter in warmer areas [6], while hibernation is longer in the north due to the longer-lasting winter and low temperatures, therefore shortening the period of time during which hedgehogs are active [3,5]. Hibernation may also be affected by local fluctuations in the weather in the given period, as has been described in Denmark, for example, where the hibernation of hedgehogs was delayed by unusually warm weather in a particular year [7].

The timing of reproduction in European hedgehogs is also associated with the timing and length of hibernation. Hedgehogs begin to mate soon after awakening from hibernation if temperature conditions are optimal, leaving plenty of resources for the hedgehogs [8], and the body conditions of the females are sufficient. Female European hedgehogs appear to be seasonally polyoestrous [5], with a succession of oestrus cycles during spring and summer. Gestation in female European hedgehogs lasts approximately 34 days, after which usually 4–5 young are born [3], with previously documented examples of litter sizes of up to 11 individuals [9]. The young are dependent on their mother’s care for a period of 5–6 weeks and are subsequently weaned at a weight of 250 g [10]. This is followed by the challenging time when the newly independent juveniles must manage to forage, with a diet comprised primarily of invertebrates, and obtain adequate day and night nests. The timing of birth influences the mortality rate in hedgehog young [11], which amounts to as much as 69% of offspring in the wild [3,12,13,14]. In the Czech Republic, hibernation ends in April and the first litters of European hedgehogs are usually born between May and August [15].

Usually, female hedgehogs have a single litter a year in areas with a colder climate, potentially enabling them to have a longer hibernation period [16]. When autumn has a high abundance of resources available, second litters may be produced in a given year [3,4,5]. Females that lose the young from the first litter may also have a second litter. They will go into oestrus again and mate, and wean their young later [10]. Offspring from second or later litters may be at a disadvantage over earlier-born young as they have less time to grow and obtain the necessary body condition before hibernation. However, Bunnell [10] studied juvenile hedgehogs taken into care—81 individuals from early litters and 38 from late litters—and demonstrated that young born in the late summer compensated for this delay by gaining weight faster than young from normally timed litters during their time in captivity with ad libitum food available [10]. It is, however, true of all young that they may find themselves in a situation in which they will not be able to survive without human help. This particularly applies to young that lose their mother before weaning. Such a scenario is sadly common as hedgehogs often live in the vicinity of towns and villages [17] and are exposed to many risks associated with anthropogenic activities, such as collisions with vehicles on the roads [18], being attacked by pets or dying as the result of injuries caused by garden tools and machinery, or from poisoning [3,4,19,20,21,22,23]. Wildlife rehabilitation centres can play an irreplaceable role in caring for individuals and therefore supporting the protection of this species [21,24]. Orphaned juvenile hedgehogs found by humans are often taken to wildlife rehabilitation centres where they are hand-raised with the aim of being subsequently released back into the wild. Previous research comparing the post-release survival of hand-raised and wild, juvenile hedgehogs has shown that hand-raised juveniles appear to have equal prospects as wild, suggesting that hand-raising of orphaned juvenile hedgehogs is an important contribution to the conservation of this species [13].

The aim of this study was to categorise the groups of juvenile western European hedgehogs (*Erinaceus europaeus*) admitted to wildlife rehabilitation centres in the Czech Republic before weaning and after weaning in the years 2011 to 2020, and to determine whether the timing of their birth (normally timed or late litters) influenced their survival chances in care. In addition, interpreting the results in a wider context to discuss how to optimise the conservation effort based on the hand-raising of orphaned, sick or injured juvenile hedgehogs.

## 2. Materials and Methods

Data on juvenile western European hedgehogs (*Erinaceus europaeus*) admitted to wildlife rehabilitation centres in the Czech Republic were obtained from the Ministry of the Environment, which is responsible for the work of wildlife rehabilitation centres and keeps records on the animals for which these facilities care. As these rescue centres are part of the National Network of Rescue Centres, which cooperates with the Ministry of the Environment, the recording system is unified. The data include information on the number of admitted animals, the dates and reasons for admission, and voluntary information on the weight or age of the animal and the place of finding if it is known. These records contained data on the numbers of juvenile European hedgehogs admitted from 2011 to 2020, the reasons for their admission to wildlife rehabilitation centres, and the dates on which these juveniles were admitted to and the outcome, e.g., whether they were released back into the wild or died (naturally or euthanised). A total of 27 wildlife rehabilitation centres in the Czech Republic provided data for the study. Identification of admitted species, including distinguishing between the two native hedgehog species of the Czech Republic (*Erinaceus europaeus* and *E. roumanicus*), and recording characteristics of each individual, were the responsibility of the rehabilitation centres’ certified experts.

### 2.1. Categorising Data

We included data on reason for admission into care and weight at admission for juvenile European hedgehogs for the purpose of this study. The juvenile hedgehogs were divided by weight at admission into the categories pre-weaned (1–250 g) and post-weaned (251–400 g) young. Furthermore, the juvenile hedgehogs were divided by date of admission into the categories “young from normally timed litters” (young admitted from April to September) and “young from late litters” (young admitted from October to March). This division was based on calculation of the gestation period (5 weeks) and the age of weaning (5–6 weeks) according to Bexton [22]: young from normally timed litters came from hedgehogs that mated during March and April at the earliest, while young from late litters came from litters for which hedgehogs had mated during August and September at the earliest, i.e., in the late summer. A weight of 250 g is stated by Bunnell [10] as the weight of hedgehogs at weaning. The individuals were divided into four groups for the purposes of comparison: pre-weaned from normally timed litters (NL PRE), post-weaned from normally timed litters (NL POST), pre-weaned from late litters (LL PRE) and post-weaned from late litters (LL POST). In some cases, information on the outcome of the care (died naturally/euthanised or released back into the wild) was lacking, which resulted in the exclusion of these records from the data analyses on survival and duration of time in care.

For the young with known outcome, the proportion of young released after care and young that died or were euthanised during care was calculated to establish the release rate and mortality rate: the proportion of juveniles released and juveniles that died or were euthanised in care compared to the total number of hedgehogs admitted in the given group (NL PRE, NL POST, LL PRE, LL POST). Out of the 4388 juvenile hedgehogs registered in the records, only 3441 individuals had information on the outcome of the care (died naturally/euthanised or released back into the wild). Therefore, the remaining 947 individuals were excluded from the data analyses on survival and duration of time in care. 

The duration of care at the wildlife rehabilitation centres was analysed for individuals that were subsequently released back into the wild, in the cases where date of admission and date of release were stated in the records. The duration of care was calculated as the number of days between these two dates.

### 2.2. Data Analysis

Statistical analysis was performed in the statistical program UNISTAT 6.5 for Excel (Unistat Ltd., London, UK). The data according to the normality test have a non-normal distribution, thus non-parametric tests were used. Spearman’s coefficient was used to assess the trend in the number of young admitted in a given period, and a Fisher’s exact test was used for evaluation of the difference in the numbers of young admitted in individual groups (NL PRE, NL POST, LL PRE and LL POST) and for comparison of the numbers of young released and young that died or were euthanised. Kruskal–Wallis ANOVA was used for the assessment of the duration of admission to the wildlife rehabilitation centre. In the tests used, the value of *p* < 0.05 was determined to be statistically significant.

## 3. Results

**Figure 1:**
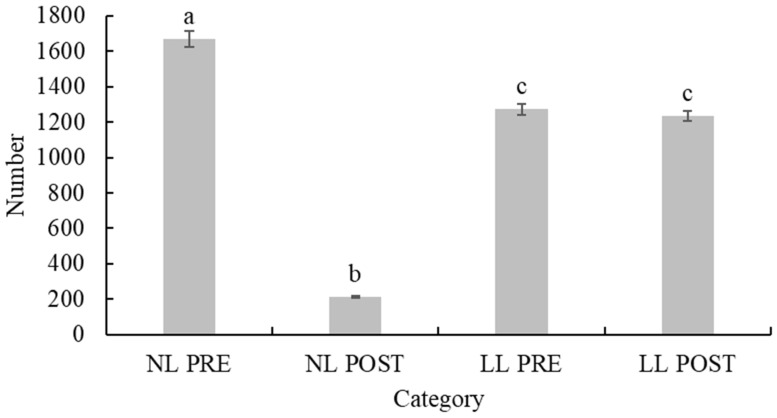
The number of juvenile European hedgehogs admitted to wildlife rehabilitation centres in the Czech Republic in the years 2011 to 2020, total 4388, divided into four categories by weight and timing of litter. Columns or bars with different letters (i.e., a versus b, a versus c, b versus c) indicate that they are statistically different from each other, while those with same letters (c versus c) indicate that they are not statistically different from each other. NL PRE = pre-weaned juvenile hedgehogs from normally timed litters; NL POST = post-weaned juvenile hedgehogs from normally timed litters; LL PRE = pre-weaned juvenile hedgehogs from late litters; LL POST = post-weaned juvenile hedgehogs from late litters.

A total of 4388 juvenile European hedgehogs were admitted to 27 wildlife rehabilitation centres in the Czech Republic in the period from 2011 to 2020, with an increasing trend in the number of juveniles admitted per year during the entire period (rSp = 0.9879, *p*< 0.001). A significantly lower number (*p* < 0.001) of NL POST (post-weaned individuals from normally timed litters) young were admitted in comparison with all the other groups (Figure 1). 

The lowest mortality rate was found in NL POST juveniles (14% of the number of animals admitted in this category), for which the highest release rate was also found (86%) (Table 1.). In contrast, the highest mortality rate was seen in the category LL PRE (46%), which therefore also had the lowest release rate (54%). There was no significant difference (*p* > 0.05) in mortality rate and release rate between NL PRE and LL POST.

**Figure 2:**
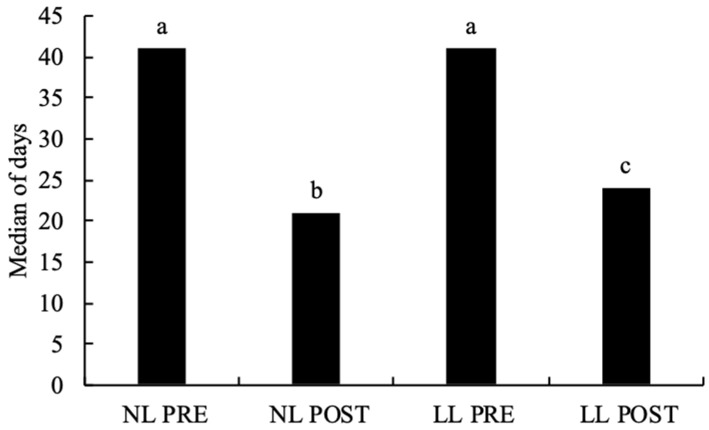
Period of time spent by released juvenile European hedgehogs at wildlife rehabilitation centres in the Czech Republic from 2011 to 2020 divided into four categories by weight and timing of litter (data are from the 3441 individuals with known outcome). Note: columns or bars with different letters (i.e., a versus b, a versus c, b versus c) indicate that they are statistically different from each other, while those with same letters (c versus c) indicate that they are not statistically different from each other. NL PRE = pre-weaned juvenile hedgehogs from normally timed litters (n = 806); NL POST = post-weaned juvenile hedgehogs from normally timed litters (n = 151); LL PRE = pre-weaned juvenile hedgehogs from late litters (n = 525); LL POST = post-weaned juvenile hedgehogs from late litters (n = 443).

The length of stay in NL PRE (median 41 days) and LL PRE (median 41 days) young was significantly longer (*p* < 0.001) than in NL POST and LL POST young (median 21 and 24, respectively) (Figure 2).

## 4. Discussion

The number of European hedgehogs admitted to wildlife rehabilitation centres has been on the increase in recent years, with their admission to wildlife rehabilitation centres in the UK doubling in the period 2005–2017 [19]. The offspring of wildlife make up a large proportion of all sick, injured and orphaned individuals that are admitted to these facilities [24,25]. Young accounted for a proportion of almost 20% of all hedgehogs admitted to three wildlife rehabilitation centres in Spain in the years 2009–2013 [20] and almost 60% in 34 wildlife rehabilitation centres in the Czech Republic from 2010 to 2019 [21].

### 4.1. Distinguishing the Species in Care

The Czech Republic is inhabited by two species of hedgehogs, the European hedgehog (*Erinaceus europaeus*) and the northern white-breasted hedgehog (*E. roumanicus*), which can be challenging to distinguish from each other, as they share a range of similar features. Our study focuses exclusively on *Erinaceus europaeus*. Our data derives from the records of 27 wildlife rehabilitation centres, where the carers have identified the individuals as European hedgehogs. Even though this categorisation cannot be validated, we acknowledge that all wildlife rescue centre staff in the Czech Republic must have a certificate from the Ministry of the Environment, which also includes an exam on identifying species living in the Czech Republic. In the case of *E. europaeus*, this includes distinguishing them from *E. roumanicus* based on morphology.

### 4.2. Categorising Individuals into Dependent and Independent Juveniles

Due to the lack of background knowledge of the individuals, our categorisation of juveniles into dependent and independent individuals was based on weight at admission, with pre-weaned weighing ≤250 g and post-weaned >250 g. There is a general consensus in the literature on the subject that juvenile hedgehogs tend to weigh around 250 g when they reach independence. However, there may have been cases where sickness or injury have caused independent individuals to weigh less than 250 g, which would ultimately have led them to become recorded as dependent juveniles. This potential bias should be considered when interpreting the results. 

### 4.3. Independent Juveniles from Normally Timed Litters Have Better Prospects

Individuals belonging to the NL POST young category were admitted to wildlife rehabilitation centres least often and had a significantly lower mortality rate in care compared to the other categories. These individuals are adolescent hedgehogs weighing 251–400 g. They are already independent, beginning to live the solitary way of life typical of this species, and learning to forage for themselves and build nests [22]. They must also prepare themselves for the coming winter when hibernation awaits them, though in view of the appropriate timing of their birth in the summer months they have enough natural food and time to attain the necessary weight and body condition, if in otherwise good health. Therefore, their prospects are generally better compared to independent juveniles. This is also indicated by the fact that this group spent significantly shorter time in care compared to pre-weaned individuals from normally timed and late litters. It should, however, be taken into consideration that the lower sample size of this particular category of individuals, compared to the rest, may have influenced the results.

### 4.4. The Potentially Negative Consequences of Care and Alternative Solutions

Our results show that the highest proportions of juvenile hedgehogs admitted into care are dependent individuals (pre-weaned; NL PRE (38%) and LL PRE (29%)). These young are still dependent on their mother’s care. However, every small (<250 g) hedgehog should not necessarily be considered orphaned and in need of care. These results suggest that dependent juvenile hedgehogs are often admitted to wildlife rehabilitation centres regardless of the timing of the litter. The ability to identify juvenile hedgehogs truly in need of human care is important, as they may otherwise be taken to wildlife rehabilitation centres needlessly. It has been shown that the capture of wild animals may itself lead to their death due to causes like capture myopathy or infection transmitted from the other patients [13,26]. Furthermore, wild animals placed in captivity, e.g., at a wildlife rehabilitation centre, encounter a novel, confined and unpredictable environment, which often includes handling and close proximity to humans [26]. These conditions cause physiological stress responses in a range of species and these increased stress levels may have severe effects on their health [13,27,28]. Rasmussen et al. [13] demonstrated that hedgehogs in care experience higher levels of faecal corticosterone metabolites and saliva corticosterone compared to wild individuals, suggesting that hedgehogs in care do experience higher levels of stress than their wild counterparts. Therefore, if a juvenile hedgehog seems to be healthy and mature enough to provide for itself, despite a small body size, human help in the form of supplementary feeding and provision of good nest sites in situ, should be recommended as an alternative to captivity. 

### 4.5. When to Admit Juveniles into Care and When to Release Them

Several signs can be recognised in pre-weaned young indicating that they could need care. These include the finding of an isolated juvenile or a litter of juveniles with individuals weighing less than 200 g, being active during the day, vocalising, or having light-coloured and soft spines [23]. Such young may have lost their mother due to e.g. car collisions during the summer, when lactating females are more active and may cross roads more frequently due to the increased rate of foraging needed to cover their intensified use of resources caused by lactation [19]. They may also be less viable young that have been abandoned by their mother [29], in which case any attempt to save them may be more complicated and perhaps even counterproductive. Caring for pre-weaned young is demanding and is furthermore an economic burden for the wildlife rehabilitation centre. One principal problem lies in assuring an adequate milk diet, as is the case for the young of many other wild animal species [30]. Hedgehog milk is extremely rich in fat and proteins and has a low lactose content [31], for which reason it may be difficult to find a suitable milk substitute. Gimmel et al. [32] have drawn attention to the shortcomings of commercially manufactured mixes for European hedgehogs, though their study did not consider milk substitutes. If the milk of other mammals is to be used, Robinson and Routh [33] recommend using goat milk, initially with a syringe, until the juveniles are mature enough to feed individually from a dish. Presently, the general practice at hedgehog wildlife rehabilitation centres is to use commercially available puppy milk replacer combined with a careful monitoring of weight gain. Hand-raising of juvenile hedgehogs should only be carried out by trained hedgehog rehabilitators as it is a specialist’s task. 

The demands of hand-rearing pre-weaned hedgehog young are evidently also associated with the fact that young admitted to wildlife rehabilitation centres before weaning spent a significantly longer (*p* < 0.001) period of time at the centre than young admitted after weaning. Pre-weaned young from both normally timed and late litters spent a median of 41 days at wildlife rehabilitation centres before being released back into the wild. Hedgehog young in the wild are weaned at the age of 5–6 weeks [23]. Young are released at a similar age or an age several weeks older than the age of natural weaning depending on their age at the time of their admission and their weight before release. 

Unfortunately, not all juvenile hedgehogs admitted to the wildlife rehabilitation centres could be released back into the wild. Some died while in care and some had to be euthanised due to severe injuries or poor prognosis. Age upon admission and the timing of the litter also influenced the outcome: Although more than a 70% of young from normally timed litters admitted were released back into the wild in the same year (70% of pre-weaned and 86% of post-weaned individuals), the numbers of pre-weaned animals released from late litters were significantly (*p* < 0.001) lower (53% individuals); the results may also be affected by fewer number of admitted post-weaned hedgehogs form normally timed litters. The smaller number of individuals from late litters released may have been influenced by the fact that the date of their release from the wildlife rehabilitation centre would have fallen in the winter, i.e., during the hibernation period, leaving the registration as “unknown” in the record. Despite research demonstrating that hedgehogs can successfully be released during winter when the conditions are suitable [34], and that hedgehogs in care experience higher levels of stress which may decrease their welfare and chances of survival [13], opinions still differ between carers as to whether hibernating animals should be kept at wildlife rehabilitation centres over the winter or released in the late autumn or during winter. The question remains as to what impact stress has on animals during their time spent at a wildlife rehabilitation centre, as it may have a negative effect on the health and normal behaviour of animals [35], and whether all animals at wildlife rehabilitation centres go into hibernation. External conditions, and temperature in particular, are important to the commencement of hibernation [8]. Hibernation is often not possible, or disturbed, in excessively small juvenile hedgehogs admitted before or during the winter when they require intensive care that includes frequent weighing and observation of the animals. Nevertheless, research indicates that weighing of hedgehogs during hibernation and their possible wakening does not affect their chance of surviving hibernation as long as it is not done too frequently [36], particularly in cases in which the energy losses during such disturbance are compensated for by the provision of food that is available in sufficient quantities at wildlife rehabilitation centres. South et al. [36] also found that the duration of hibernation meant greater weight losses in smaller hedgehogs which lost weight more quickly during hibernation at the wildlife rehabilitation centre. The lower release rate in the group of LL POST individuals could also be caused by the condition of the individuals taken into care, as it is expected that individuals from later litters are generally less robust than individuals from normally timed litters due to the lack of food resources later in the season. The high release rate in post-weaned young from normally timed litters may also testify to the fact that rescue centres in the Czech Republic are in practice more likely to release young in summer and less likely to release them in winter for fear that they will not survive hibernation, even though previous studies have indicated that this is should not be a cause for concern [4,17,34,37].

### 4.6. Optimising the Care

In general, pre-weaned young from normally timed litters and post-weaned from late litters displayed similar mortality rate at wildlife rehabilitation centres in the Czech Republic; however, the highest mortality rate was found in pre-weaned hedgehogs from late litters. Mortality is generally high in animals at wildlife rehabilitation centres [24]. Furthermore, time spent at wildlife rehabilitation centres is a stress factor for hedgehogs increasing the level of glucocorticoids in the faeces and saliva in comparison with free-living hedgehogs [13] and the length of time they spend at such centres should be kept to the absolute minimum.

The results indicate that the timing of hedgehog births may also affect the chances of survival for juveniles admitted to wildlife rehabilitation centres and suggest that they may have different care requirements. Therefore, in addition to general care, the aim of wildlife rehabilitation centres should be to develop procedures and methods to care for hedgehogs at different ages and consider whether the effort should be adapted according to the period during which they were born and admitted to wildlife rehabilitation centres. Another task should be to optimise releases into the wild to ensure the highest possible survival rate after release and not to unnecessarily prolong the stressful stay in wildlife rehabilitation centres for these animals.

### 4.7. The Influence of Members of the Public on Admissions of Juvenile Hedgehogs

The public plays a large role in the admission of animals to wildlife rehabilitation centres by reporting findings of animals requiring human care to the staff of these centres or by bringing sick, injured or orphaned animals to the centres. Fewer NL POST than NL PRE juvenile hedgehogs were admitted to rescue centres, which may be due to public education stating that only young weighing less than 250 g that are found alone may need help, as opposed to young weighing more than 250 g. The situation may be different for young found in autumn, which raises concerns in people as to whether the juvenile hedgehog will be able to put on the necessary weight needed for hibernation. The results indicated a significantly (*p* < 0.001) lower proportion of NL POST hedgehogs admitted than LL POST. We suggest that even though these animals may have the same weight, the juveniles from late litters are considered more at risk by the public because of the approaching winter. This is causing members of the public to regard even large, healthy and independent juvenile hedgehogs as threatened and requiring human care simply because they remain active in the late autumn. The admission of seemingly healthy individuals belonging to the LL POST category could also be explained by the inability of members of the public and even hedgehog carers to distinguish between independent and dependent juvenile hedgehogs. The law in the Czech Republic requires rehabilitation centres to provide education to the public and thus participate in the protection of species in the wild including examples of the work of these centres and information materials.

The issue of an adequate weight before hibernation is well known, and many wildlife rehabilitation centres, along with other organisations contributing to the protection of European hedgehogs and other wildlife species, issue manuals containing information about how to identify individuals that require human help. Hibernation is a demanding period during which the animal uses a large amount of energy [36]. According to Robinson and Routh [33], hedgehogs weighing less than 450 g in the autumn, found out of the nest during the day, or showing signs of weakness or health problems may require help to survive the winter. Morris [38] suggested a hibernation weight of >450 g as a threshold for winter survival if a 25% weight loss during hibernation occurs. According to Bearman-Brown et al. [39] hibernation is not a critical period for hedgehogs that hibernate at a weight of at least 600 g. Wildlife rehabilitation centres can help hedgehogs attain the necessary weight before hibernation during their time spent in captivity [40]. However, if a hedgehog is otherwise healthy, supplementary feeding and provision of suitable nest sites in situ is a much more desirable solution, avoiding taking the individual into care. Awareness of the risk of death in immature young hedgehogs during the winter may be the cause of the larger proportion (28%) of LL POST young admitted to rescue centres in the Czech Republic. Finding a young hedgehog in the autumn more often leads to its admission at a wildlife rehabilitation centre compared to the finding of a hedgehog of the same weight in the early summer. Rasmussen et al. [4] demonstrated that healthy juvenile hedgehogs are perfectly able to reach an adequate body condition to survive hibernation even when born later in the season, when resources are available. The same study also suggests directing the focus on body condition before hibernation instead of weight, as a small hedgehog of 600 g would be in good condition, while a large hedgehog of 600 g could be in a poor condition, indicating weakness of some sort, making it less likely to survive hibernation. Furthermore, previous studies exploring the body mass change in hedgehogs before and after hibernation have shown that the individuals that can afford to lose most body mass do in fact lose most body mass [3,7,37,41]. This is also related to the timing of hedgehog releases into the wild, with Yarnell et al. [34] reporting that the survival rates of rehabilitated hedgehogs released during milder periods of the winter are equal to those of wild individuals.

## 5. Conclusions

Wildlife rehabilitation centres contribute towards maintaining populations of wild species of animal by caring for sick, injured or orphaned wildlife. The reproduction of European hedgehogs in the Czech Republic is influenced by climatic conditions, and young born in normally timed and late litters may have different care demands and survival chances. Although juvenile hedgehogs from normally timed litters can be released successfully back into the wild in most cases, the mortality rate is higher in pre-weaned young from late litters at the wildlife rehabilitation centres. As female hedgehogs often give birth to two litters per year in the Czech Republic, and the population of European hedgehogs is in decline, further efforts must be made to improve the care for juvenile hedgehogs, particularly before weaning, to increase the survival, and therefore also the release rate, of these animals.

## Figures and Tables

**Table 1:** Outcome of juvenile European hedgehogs admitted to wildlife rehabilitation centres from 2011 to 2020 divided into four categories by weight and timing of litter (data are from the 3441 individuals with known outcome).

Litter Timing	Category	Admitted (Number)	Mortality Rate		Release	
			Number	%	Number	%
**Normally timed litter**	pre-weaned	1518	453 ^a^	30	1065 ^x^	70
	post-weaned	210	30 ^b^	14	180 ^y^	86
**Late litter**	pre-weaned	983	455 ^c^	46	528 ^z^	54
	post-weaned	730	251 ^a^	34	479 ^x^	66

^a–c^ different letters in a column indicate a statistically significant difference (*p* < 0.01), with fields marked with a being statically significantly different from fields categorised as b and c, whereas fields indicated with the same letter represent data which are not statistically significantly different from each other. ^x–z^ different letters in a column indicate a statistically significant difference (*p* < 0.01).

## Data Availability

Data for the analysis was obtained from the Ministry of the Environment of the Czech Republic. The datasets generated and analysed during the current study are available from the corresponding author on reasonable request.
